# A SIMPLE INTERVENTION TO REDUCE ANTICHOLINERGIC DRUG USE WHILE ATTENDING A GERIATRIC DAY HOSPITAL

**DOI:** 10.1093/geroni/igz038.2603

**Published:** 2019-11-08

**Authors:** Costa Apostolides

**Affiliations:** Dalhousie University, Halifax, Nova Scotia, Canada

## Abstract

Attendance at a Geriatric Day Hospital has previously been shown to reduce both the overall number of medications and the number of anticholinergic medications of patients. In the present study, patients enrolled in a Geriatric Day Hospital program from January to February 2019 were divided into a control and intervention group. Anticholinergic medications in the intervention group were flagged by highlighting them in the patient chart and alerting the attending clinician, whereas no alerts were provided in the control group. Anticholinergic load was calculated using the Anticholinergic Cognitive Burden (ACB) and Drug Burden Index (DBI) scores. In comparing admit versus discharge medications in the intervention group, both the mean number of overall medications (10 vs. 9.7) and anticholinergic medications (3.5 vs. 3.1) was reduced; this was not the case in the control group, where the mean number of overall medications remained the same (11.92) and the mean number of anticholinergic medications increased (3.83 vs. 3.92). More significantly, in comparing admit versus discharge scores, both the mean ACB and DBI scores were reduced in the intervention group, but in the control group both the ACB and DBI scores either remained the same or increased at the time of discharge. This clearly shows that a simple intervention (highlighting anticholinergic medications in the patient chart) can have a clinically beneficial outcome of reducing these harmful medications in patients. With approximately 50% of the older population taking at least one anticholinergic drug, the importance of reducing anticholinergic burden cannot be overemphasized.

